# Consciousness and cognitive control

**DOI:** 10.2478/v10053-008-0097-x

**Published:** 2012-02-03

**Authors:** Wilfried Kunde, Heiko Reuss, Andrea Kiesel

**Affiliations:** Department of Psychology, Julius-Maximilians-University Wuerzburg, Germany

**Keywords:** cognitive control, consciousness, priming

## Abstract

The implementation or change of information processing routines, known as *cognitive control*, is traditionally believed to be closely linked to consciousness. It seems that we exert control over our behavior if we know the reasons for, and consequences of, doing so. Recent research suggests, however, that several behavioral phenomena that have been construed as instances of cognitive control can be prompted by events of which actors are not aware. Here we give a brief review of this research, discuss possible reasons for inconsistencies in the empirical evidence, and suggest some lines of future research. Specifically, we suggest to differentiate cognitive control evoked either because of explicit or because of implicit control cues. While the former type of control seems to work outside of awareness, the latter type of control seems to be restricted to consciously registered events that call for control.

## Introduction

It has been known for a long time that unconscious stimuli can affect our behavior. Classical demonstrations of this phenomenon relate to neurological cases of blindsight, neglect, or extinction, where patients, despite being unaware of parts of their visual field, locate and identify stimuli above chance level (e.g., Fuentes & Humphreys, 1996; Pöppel, Held, & Frost, 1973; Weiskrantz, 1986, 2002; Young & de Haan, 1993). In healthy participants similar phenomena have been demonstrated by means of subliminal priming. In subliminal priming experiments, participants respond to a target that is preceded by another, so-called *prime stimulus*. Although the prime is heavily masked, and thus phenomenally “unaware”, it leaves a trace in behavior: Responses are usually faster and more accurate when prime and target are mapped to the same motor response (e.g., Dehaene et al., 1998; Neumann & Klotz, 1994; Vorberg, Mattler, Heinecke, Schmidt, & Schwarzbach, 2003) or belong to the same semantic category (e.g., Dell’Aqua & Grainger, 1999; Kiefer, 2002; Kiefer & Brendel, 2006; Martens, Ansorge, & Kiefer, 2011; Schütz, Schendzielarz, Zwitserlood, & Vorberg, 2007), which implies that the prime is processed to some degree.

Explanations of vision without awareness typically assume that even unconscious stimuli are processed, provided the cognitive system was prepared in advance to do so (e.g., Ansorge & Neumann, 2005; Kiesel, 2009; Kiesel, Kunde, & Hoffmann, 2007; Kunde, Kiesel, & Hoffmann, 2003, 2005; Martens & Kiefer, 2009; Neumann & Klotz, 1994; Pohl, Kiesel, Kunde, & Hoffmann, 2010). Thus, the effectiveness of subliminal stimuli depends on preparatory processes that occur in advance of, and set the stage for, such subliminal stimuli. There are many different versions of this basic assumption, varying for example on whether actual practice with certain stimuli is necessary for efficient preparation (e.g., Damian, 2001), or whether the mere intention to respond to these stimuli suffices (Naccache & Dehaene, 2001), or whether appropriate preparation enables semantic processing of subsequent unconscious stimuli (Dehaene et al., 1998) or remains confined to the analysis of perceptual features (Kunde et al., 2003). Despite such differences, all these models share common ground: Stimulus awareness is not necessary for processing, given that the observer/actor is prepared to encounter these stimuli (e.g., Dehaene & Naccache, 2001).

## A reservation of consciousness: Cognitive Control?

The above review shows that the processing of subliminal stimuli is widely acknowledged. In fact, sometimes the capacity for unconscious processing is assumed to be even larger than the capacity for conscious processing (e.g., Custers & Aarts, 2010; Dijksterhuis, Bos, Nordgren, & van Baaren, 2006). One may therefore wonder whether there is anything left at all that “the unconscious” cannot do. Stated conversely, are there processes that can only operate on events we are aware of? Answering this question is important because knowing which pro-cesses require awareness and which do not shed light on the functional role of awareness in human information processing. Indeed, some researchers claim that cognitive control processes obligatorily require awareness (e.g., Dehaene, & Naccache, 2001; Jack & Shallice, 2001; for an overview, see Hommel, 2007). The term *cognitive control* is widely used in modern psychology and describes phenomena that have been considered in chapters on “will” in historical textbooks of psychology (e.g., Ach, 1905). Although not very well defined, it seems fair to say that *cognitive control* denotes those processes that configure the cognitive system to process stimuli in a specific manner, and re-configure the cognitive system when certain events tell the observer/actor to treat stimuli in a different way.

There are many different situations or experimental effects that are assumed to include such control operations. For example, in studies on task switching, participants have to respond to the same stimuli in a different way depending on the currently instructed task context, and thus cognitive control is believed to be heavily involved in task switching (Kiesel et al., 2010; Monsell, 2003). Likewise, in stop-signal tasks, a sudden stimulus tells the actor to inhibit the response to a stimulus (Logan, 1982). Here the assumption is that the “cognitive veto” (to not carry out the response that would otherwise occur) is an instance of cognitive control. Another proposed instance of cognitive control is the adaptation to conflicting response tendencies that are based on automatic processing of irrelevant information. Such conflicting tendencies are a hallmark of so-called *interference tasks*, such as the Simon task (Simon, 1969), the Eriksen task (Eriksen & Eriksen, 1974), or the Stroop task (Stroop, 1935). In these tasks, a nominally task-irrelevant stimulus or stimulus feature suggests a different response than is actually required. The crucial observation is that when response-conflict is generally frequent (e.g., in a block of trials), or has just been expe-rienced (e.g., in the preceding trial), the information processing is altered in subsequent trials, such that, for example, more attention is devoted to task-relevant rather than to task-irrelevant stimulus aspects. As a consequence of this control impact, the influence of irrelevant information with frequent or recent response conflict is reduced (Botvinick, Braver, Barch, Carter, & Cohen, 2001; Gratton, Coles, & Donchin, 1992; Kiesel, Kunde, & Hoffmann, 2006; Kunde, 2003; Kunde & Wühr, 2006), and responses generally slow down (Kiesel et al., 2006; Verguts, Notebaert, Kunde, & Wühr, 2011).

 Regarding the interplay of consciousness and cognitive control, it is important to note that we are normally aware of the events that call for a modification of our routine actions. These may be external events such as changes in the environment, warning signals, the occurrence of errors, or internal events such as experienced effort in selecting appropriate behavior or some kind of self-instruction (Goschke, 2000). Intuitively, there is a strong link between awareness and the recruitment of cognitive control. Also, in psychological theorizing, cognitive control processes are strongly related to consciousness, in the sense that these processes rely on conscious decision-making (Dehaene & Naccache, 2001; Jack & Shallice, 2001; Umilta, 1988; for an overview, see Hommel, 2007). For example, Jack and Shallice (2001) emphasize that the underlying processes engaged by conscious action are different from those engaged by automatic action. Similarly, Dehaene and Naccache (2001) suggest in their workspace model that routine actions are possible without consciousness, while consciousness is required for cognitive control. They state that “it should be impossible for an unconscious stimulus to modify processing on a trial-by-trial basis through top-down control“ (Dehaene & Naccache, 2001, p. 21) and that “an unseen prime cannot be used as a source of control to modify the choice of processing steps“ (Naccache, Blandin, & Dehaene, 2002, p. 423). 

Although such a control-consciousness link appears persuasive, it is a matter of empirical research to determine whether it holds true. First of all, it is important to scrutinize the problem under investigation. The questions are not whether observers/actors become aware that events exert cognitive control and how this happens. The answers to those questions are likely negative. Normally, we barely notice that we do something like “focusing attention” or “activating a task set”. We conjecture that cognitive control does not require meta-knowledge of when and how to apply it. Instead, we believe that the important and empirically testable question is whether changes of behavior through cognitive control occur when the control-invoking event remains unnoticed.

We will review a substantial number of studies that pursued this research strategy. To anticipate a main result, the outcome of this research program is ambiguous. Some studies suggest that cognitive control can be prompted by events that remain unnoticed, whereas other studies suggest that they cannot. This apparent discrepancy calls for clarification. A first step towards such clarification is to sort the evidence into cases where unconscious invoking of control is consistently found and those where it is not. One possible categorization concerns the types of events that call for control. These events vary enormously regarding complexity, duration, ambiguity, and so forth. We found it most favorable to arrange the presentation of studies according to a distinction in two types of control-invoking events, namely explicit and implicit events.

*Explicit events* consist of one distinct stimulus that directly informs the observer what is requested from him or her. This applies, for example, to a stop signal. In stop-signal tasks, an explicit stop signal instructs to withhold an already selected motor response (Logan, 1982). Likewise, in an explicit task cuing procedure, a task cue instructs the actor which stimulus-response mapping to apply, and which aspect of the stimulus to attend to in the next trial (Meiran, 1996). Thus, a stop signal is linked to the termination of response execution and a task cue requires the recollection of a certain task set.

In contrast, *implicit events* encompass more than one single, explicit stimulus and they have to be derived from the task environment. This applies, for instance, to frequency manipulations across a block of trials, such as a manipulation of the frequency of conflict-laden trials in an experimental block of a Stroop task (e.g., Logan & Zbrodoff, 1979). The control-invoking event in this case (the proportion of incongruent trials) is an abstract property of several preceding episodes which are spread in time. Moreover, the appropriate consequence of encountering such an event, such as “focusing on task-relevant stimuli,” is less clearly defined than in the explicit case. The explicit-implicit distinction is an empirical one, but it may relate to functional differences as well, which we will discuss later. In the following, we first discuss control invoked by explicit events, since the situation is relatively undisputed in this case.

## Cognitive control invoked by explicit events

To our knowledge, there are three instances of cognitive control that have been tested to be subliminally induced by explicit cues. These are task preparation, response inhibition, and orienting of attention.

### Task preparation

 Several studies investigated whether masked primes have the power to activate task sets. Mattler (2003, 2005, 2006, 2007) used a task switching paradigm in which a cue preceded an imperative stimulus. The cue informed the participants as to which task should be performed on a subsequent stimulus. For example, the cue informed whether the pitch or the timbre of a tone should be judged. In Mattler’s studies, the task cues were preceded by a masked, and hence invisible, prime. Importantly, in trials in which the prime resembled the task cue, performance was facilitated, suggesting that the prime prompted the pre-paration of the corresponding task. Alternative explanations, such that the prime merely facilitates the perceptual encoding of the subsequent task cue rather than activating the task set, were ruled out by using se-veral perceptually dissimilar cue exemplars (Mattler, 2006). Later, Lau and Passingham (2007) observed that the masked primes also activate brain areas that are known to be involved in performing the respective tasks. Reuss, Kiesel, Kunde, and Hommel (2011) complemented these findings by showing that not only is the switching towards a cued task facilitated by masked primes, but also that masked primes determine which task participants prefer to carry out. Participants in that study were shown task cues that told them which of two tasks (e.g., judging parity or magnitude of a digit) they should perform on a later presented target stimulus, or cues that told them whether to repeat the current task or switch to the other one. Sometimes the cue was masked and thus participants had the free choice to carry out whichever task they wanted. In these situations, the subliminally cued task or the subliminally cued task repetition/alternation was preferred. Thus, a masked stimulus does not only facilitate task preparation, but it may also trigger which task is eventually selected. In sum, there is little doubt that masked stimuli have the power to induce task sets. 

### Response inhibition

 Another, perhaps even more basic, cognitive control process is the inhibition of motor output. Such inhibition is particularly challenging when a motor action is already prepared and is about to be executed. These conditions are fulfilled in stop signal experiments (Logan, 1982). In such experiments, participants carry out speeded responses to certain stimuli. In some occasions a stop signal is presented after the imperative stimulus that asks the participants to withhold the already prepared response. The assumption is that in such cases inhibitory control, that is a cognitive veto, is needed to shut down motor output. This task was used by van Gaal, Ridderinkhof, van den Wildenberg, and Lamme (2009) . The innovative modification in their experiment was that the stop signal was sometimes masked so that participants had no clue that it had been presented. Even after an invisible stop signal responses were sometimes fully inhibited, and responses were delayed in cases where they were not inhibited. The authors therefore concluded that a masked stimulus can invoke inhibitory control processes. In another study, van Gaal, Lamme, and Ridderinkhof (2010) reported that the efficiency to inhibit responses after masked stop signals correlates with the amplitude of the N2 of the brain activity that was time-locked to the stop signal. It is possible that this brain potential signals the start of the inhibition process. Similar inhibition effects were reported for Go/Nogo tasks (Hughes, Velmans, & De Fockert, 2009; van Gaal, Ridderinkhof, Fahrenfort, Scholte, & Lamme, 2008). Here, participants have to respond to a certain Go-stimulus, and they must refrain from responding in trials in which a Nogo stimulus appears. Response time is delayed when a Go stimulus is preceded by a masked version of a Nogo stimulus rather than by another neural stimulus, which suggests that the subliminal Nogo event triggers to some extend response inhibition. 

 Interestingly, van Gaal et al. (2009) observed that another instance of cognitive control, post-error slowing, did not occur. In stop signal tasks, responding in Go trials is slowed down if the participant failed to inhibit a response in the previous trial (e.g., Rieger & Gauggel, 1999). In the study of van Gaal et al., this post-error slowing effect occurred after failures to inhibit responses when a visible stop signal was presented, yet it did not occur when the stop signal was masked. In the same experiment, one type of control (the inhibition of responses) did occur without awareness of the control-invoking event (the stop signal), whereas another instance of control (post-error slowing) did not occur without awareness of the invoking event (an erroneous response). The authors conclude that their results “converge with studies showing that awareness seems crucial for some types of (trial-by-trial) cognitive control regulations … but also demonstrate the possibility of unconsciously triggered inhibitory control” (p. 1136).

### Orienting of attention

 While response inhibition can be described as cognitive control at the output side, there is also cognitive control at the input side, namely in the selection of stimuli. For example, humans can deliberately attend to stimuli in one modality, such as vision or audition. Mattler (2003) cued participants as to whether they should respond to stimuli in either the visual or auditory modality while presenting different stimuli in both modalities simultaneously. The modality cue was preceded by a masked prime that was perceptually similar either to the cue for the visual or to the auditory modality, meaning the masked prime and the cue could either signal the same modality or different modalities. Performance was superior if the prime signaled the modality that participants were required to attend to, according to the subsequent cue. This led Mattler to conclude that the subliminal prime already produced an orienting of attention to the corresponding sensory modality. 

 Within the visual modality humans can orient attention covertly to different locations of their visual fields even without moving their eyes. This can happen due to a sudden change in the environment that automatically draws attention to that location (Jonides & Yantis, 1988), or because a certain symbolic cue predicts where in the visual field a relevant target is to be expected (Posner, 1980). A number of studies demonstrated that unconsciously presented exogenous cues induce covert shifts of attention concerning this first type of attention orienting, the exogenously driven attention (e.g., Ansorge & Heumann, 2006; Ansorge & Neumann, 2005; Ivanoff & Klein, 2003; Lambert, Naikar, McLachlan, & Aitken, 1999; McCormick, 1997; Mulckhuyse, Talsma, & Theeuwes, 2007; Scharlau, 2002; Scharlau & Ansorge, 2003; Scharlau & Neumann, 2003; for a review, see Mulckhuyse & Theeuwes, 2010). Regarding the consciousness-control issue, the second type of attention orienting that is proposed to occur in an endogenously controlled manner is particularly interesting. Reuss, Pohl, Kiesel, and Kunde (2011) found that masked arrow cues did in fact facilitate the processing of targets in the cued location. But they did so only when masked cues occurred in a context of visible cues that were predictive for the target location. The authors concluded that it is only when observers have the intention to use the cues that masked versions of such cues prompt shifts of attention. 

To summarize, in the studies reviewed thus far the need for cognitive control is conveyed by a distinct stimulus. Moreover, participants had practice with visible versions of these stimuli. If such conditions are met, subliminal exemplars of these stimuli produce behavioral effects that can be considered as instances of cognitive control.

## Cognitive control invoked by implicit events

### Conflict frequency

 The need for control is sometimes not explicitly signaled, but is merely implicitly suggested, by the environment. One intensively studied control phenomenon of this type is the adaptation to conflict (Egner, 2008). As already briefly explained, conflict typically occurs in so-called *interference tasks*. Interference effects show that the human observer cannot be entirely shielded against processing irrelevant information. However, the extent to which irrelevant information is processed depends on the experienced utility of that information. For example, when response conflict occurs frequently in an experiment, inter-ference effects decline, suggesting that the processing of irrelevant input is reduced (e.g., Funes, Lupiáńez, & Humphreys, 2010). In fact, when the irrelevant information more often suggests the incorrect rather than the correct response, interference effects can even reverse (i.e., faster responding in incongruent rather than congruent trials). Apparently, observers then use the irrelevant information to strategically prepare a response that is *not* suggested by irrelevant information but which will probably be correct (Logan & Zbrodoff, 1979). Importantly, such strategic adaptation to conflict frequency occurs only when the irre-levant information is consciously perceived (e.g., Cheesman & Merikle, 1985; Merikle & Joordens, 1997; Merikle, Joordens, & Stolz, 1995). For example, Merikle and Joordens (1997) presented primes in a variant of the Stroop task for a longer or shorter duration so that the primes were either clearly visible or essentially invisible. The participants adapted to conflict frequency and responded faster on incongruent trials than on congruent trials. However, this occurred only when the primes were presented for the long duration and were thus visible. When the primes were presented for a shorter duration, and were thus invisible, the regular Stroop effect was observed with faster responding in congruent rather than incongruent trials. 

The manipulation of prime visibility by manipulation of prime duration was not optimal, since this also changed the stimulus-onset asynchrony (SOA) between prime and target, which in itself can result in reversed congruency effects (Eimer & Schlaghecken, 1998). However, predictive unconscious invalid primes have also turned out to be inefficient with constant timing parameters (Ansorge, Heumann, & Scharlau, 2002, Experiment 3).

These observations suggest that strategic changes of information processing do not occur in adaption to manipulations of conflict frequency of which the actor is not aware. Related observations have also been made regarding the manipulation of perceptual format of stimuli. While observers adapt to frequency manipulations of the perceptual format of visible targets (specifically, whether numerical stimuli are presented as digits or number words), no such adaptation occurs when format frequency is manipulated in invisible primes (Van den Bussche, Segers, & Reynvoet, 2008).

Nonetheless, the evidence regarding adaptation to conflict frequency is not unequivocal. Jaśkowski, Skalska, and Verleger (2003, Experiment 2) found that masked priming effects declined from a condition with 80% congruent trials to a condition with 20% congruent trials. However, the authors consider this effect to not be a direct consequence of conflict frequency. Rather, they propose that “effects of subliminal priming are under observers’ strategic control, with the criterion presumably set as a function of the openly observable error frequency” (p. 911). In other words, the adaptation to (unconscious) conflict frequency is considered to be mediated by adaptation to (conscious) error rates. Adaptation to error frequency might also explain congruency proportion effects with masked primes in a study by Klapp (2007, Experiment 3).

A related explanation applies to a series of studies by Bodner and colleagues where congruency proportions have been found to shape congruency effects in masked priming (Bodner & Dypvik, 2005; Bodner & Masson, 2001, 2003; Bodner & Mulji, 2010). In some experiments objective measures of prime visibility were missing (Bodner & Masson, 2001), or they revealed above-chance prime-discrimination performance (Boder & Dypvik, 2005) which makes it hard to judge the role of prime awareness for the observed effects. Even if we set the notorious problem of prime visibility aside, alternative explanations have been proposed that do not interpret this effect to be a direct adaptation to unconscious conflict frequency. The ASE (adaptation to the statistics of the environment) model proposed by Kinoshita, Mozer, and Foster (2008) explains these effects as adaptation to trial difficulty rather than to prime usefulness. Blocks with many incongruent primes are more difficult than primes with many congruent primes. Participants may notice subtle difficulty differences and then adapt to them (for a more detailed explanation along these lines, see Van den Bussche & Reynvoet, 2008).

### Context-specific conflict frequency

 Recently, another instance of cognitive control has been proposed, namely adaptations to context-specific variations of conflict frequency. The crucial observation is that congruency effects decline in perceptual contexts in which interference is high (high proportion of incongruent trials), and increase in contexts in which interference is low (low proportion of incongruent trials, Corballis & Gratton, 2003; Crump, Gong, & Milliken, 2006; Lehle & Hübner, 2008; Vietze & Wendt, 2009). The context is normally a task-irrelevant feature, such as the presentation location, that varies unpredictably from trial to trial, and that is presented only briefly before or simultaneously with the imperative stimuli. Adaptations to context-specific conflict frequency are striking because they suggest a very high flexibility and speed of cognitive control operations that affect response. The question again is whether such context-specific adaptation effects occur even when response conflicts are induced by subliminal primes, so that no representations of context-specific conflict frequencies can evolve. To study this, Heinemann, Kunde, and Kiesel (2009) used a subliminal priming task. Participants had to categorize a target number as being smaller or larger than 5. The target was preceded by a prime number that was also smaller or larger than 5. In Experiment 1, these primes were masked rather weakly (vi-sible primes). Each trial started with the presentation of a fixation cross surrounded by a colored rectangle. The color of this rectangle was the context. For one color, trials were mostly (80%) congruent (low-interference context), whereas with the other color trials were mostly (80%) incongruent (high-interference context). With visible primes, participants adapted to the context-specific conflict proportion. The congruency effect amounted to 54 ms in the low-interference context, and to 32 ms in the high-interference context. Because the size of the congruency effect is a measure for the influence of interfering information, the context-specific congruency effect shows that context information was used to inhibit prime processing in the high-interference context or, alternatively, to facilitate prime processing in the low-interference context. With subliminal primes (Experiment 2) participants showed an overall congruency effect of similar size as with supraliminal primes. Importantly in contrast to the results of Experiment 1, this congruency effect was not affected by context information. 

This finding qualifies observations by Crump and colleagues (Crump et al., 2006; Crump, Vaquero, & Milliken, 2008). Crump et al. showed that global knowledge about the frequency of congruent and incongruent events in different contexts is no pre-requisite for context-specific proportion congruency effects (CSPC effects). The amount of explicit knowledge of such congruency imbalances did not correlate with CSPC effects, nor did such knowledge boost CSPC effects. Thus, the learning processes that bring about CSPC effects require sufficiently strong (i.e., conscious) codes of prime, target, and context but they need not end in, or depend on, explicit knowledge of context-specific congruency proportions.

### Conflict recency

Another currently intensively studied trace of cognitive control is adaptation to recently experienced response conflict. The typical finding, sometimes called the Gratton effect (Gratton et al., 1992), is a reduction of congruency effects in trials that directly follow an incongruent (conflict-laden) trial in interference tasks. The phenomenon as such has been replicated many times for various interference tasks (see e.g., Egner, 2008, for a recent review). The common explanation is that experiencing conflict helps to overcome later conflict by invoking control mechanisms that amplify the processing of relevant information (Egner & Hirsch, 2005) or attenuate the processing of irrelevant information (e.g., Botvinick et al., 2001).

 The important question in the present context is: Do such conflict adaptation effects occur, even when participants are not aware of a conflict in the preceding trial? A paradigm that is suited for studying this question is, again, the masked priming paradigm, because the potentially interfering information (i.e., the prime) can be masked efficiently. Over the last 15 years there have been several investigations of this issue. The first study was reported by Greenwald, Abrams, and Draine (1996). These authors found a conflict adaptation effect in conditions with clearly visible primes, but no such effect with masked primes. This finding was replicated by Kunde (2003) . That study contained an experiment with prime visibility manipulated trial-by-trial. Interestingly, the conflict adaptation effect did not occur when the just-preceding trial contained an invisible prime. It did occur, however, when the just-preceding trial contained a visible prime, even when the prime in the current trial was invisible. This observation implies that prime visibility is necessary to invoke cognitive control, whereas the control processes that alter information processing can operate on masked stimuli as well. A problem of both the study by Greenwald et al. (1996) and of Kunde (2003) is that prime visibility was manipulated by variation of prime presentation duration, whereby possibly not only prime visibility, but also conflict size, varied. However, in a recent study by Frings and Wentura (2008) the data pattern was replicated despite identical prime target intervals and, importantly, almost identical basic interference effects for visible and invisible primes. Another replication was provided by Ansorge, Fuchs, Khalid, and Kunde (2011) . In that study, participants were asked after each individual trial, whether they believed that the preceding trial contained an incongruent prime or not. Conflict adaptation occurred when the prime in the preceding trial was clearly perceptible. When the prime in the preceding trial was not perceptible, no conflict adaptation occurred, even if participants accidentally judged the (in)congruency of that prime correctly. Apparently, only the actual experience, not the mere conjecture of conflicting information, prompts conflict adaptation. 

 Again, the evidence is ambiguous. Van Gaal et al. (2010) reported a Gratton effect even when primes were heavily masked. The procedure was almost identical to that of the study by Kunde (2003) , except for the omission of a brief warning signal at the beginning of each trial and slightly longer trial durations. At present it is not clear whether these apparently minor procedural differences were really crucial. However, if they were, this might point to a role of some kind of memory of the previous trial. Possibly, such memories are weaker with a masked ra-ther than unmasked prime, and more strongly affected by an interfering warning signal and increased trial durations. 

## Discussion

What can we learn from this review of studies on the consciousness-control link?

### Explicit and implicit events

The distinction between explicit and implicit control-invoking events we suggest here is an empirical one. However, this distinction reveals a relatively clear pattern. If the need for control is conveyed by an explicit event, awareness of that event is dispensable. However, if an event such as recent or frequent response conflict merely implicitly suggests the need of cognitive control, awareness of this event seems essential, or at least, evidence for the induction of control phenomena without awareness is not consistently found. At present we see two plausible explanations.

First, the implicit events we considered here (conflict that occurred recently, frequently, or context-specifically) conceivably all require some sort of memory. For example, for a previous incongruent trial to affect processing in a subsequent trial, some trace of that previous trial is necessary. Only conscious event representations might be strong enough to bridge longer time intervals. This is even more important when it comes to adapting to statistic manipulations such as conflict frequency and context-specific variations of conflict frequency. In these cases information of several such events has to be accumulated over a long period of time, and over a certain number of such events to extract, for example, the proportion of incongruent trials. In contrast, the typical time course with explicit control invoking events demands no accumulation of knowledge. Here, the time interval between the occurrence of a certain instruction stimulus (e.g., a task cue or a stop-signal), and the point in time when the impact of cognitive control becomes apparent (mostly by responses to a certain stimulus) is barely longer than a few hundred milliseconds, and accumulation of know-ledge over several explicit events is not necessary.

Second, as noted in the Introduction, it is a prominent idea of se-veral theories of action control to assume that stimuli instantaneously and unconsciously affect behavior only when specific if-then-plans exist (Bargh, 1989; Gollwitzer, 1999; Hommel, 2000; Kunde et al., 2003; Neumann & Klotz, 1994). This idea has fared pretty well when it comes to explaining the activation of relatively simple behaviors (keypressing, in most cases). However, it is not far-fetched to construe a cognitive control process as a kind of “response” of the cognitive system to certain stimuli, namely the control-invoking events we considered here. For example, the control process to inhibit a response or to implement a task set according to an explicit cue might be specified as the plan “if stimulus/cue X, then inhibit each response / activate task set Y”. It is known that if-then plans fail when either the “if”-side, that is the description of crucial events, or the “then”-side, that is the description of what to do, are not sufficiently specific (Gollwitzer, 1999). With explicit events participants have ample opportunity to shape their if-then plans. Participants see the stimuli and they are told what to do, and can even practice these plans before they encounter masked versions of the stimuli. The conditions are less favorable with implicit events. The “if“-part of such plans are defined rather vaguely: “Response conflict”, for instance, is something we have no sensory organ for, but we have to realize that there is conflicting information that suggests different response alternatives. The “then”-part of the plans, the “what to do”-part, is also not very clear. What might be appropriate means to adapt to response conflict? It seems that, for example, adaptation to response conflict requires a specification or correction of if-then plans, and perhaps it is exactly this alteration of plans that requires awareness.

### Lines of future research

What is important for future research on the control-consciousness link? First, we think it is worthwhile to further corroborate the proposed separation of explicit and implicit control-invoking events. If we take as a conclusion of this review that control effects following explicit events are consistently observed, while control effects following implicit events are not, two questions arise:

1. What are the limits of explicitly prompted control, and what are the necessary preconditions of implicitly prompted control?

The first question relates to the sorts of cognitive control effects that may be subliminally activated. Task switching, response inhibition, and orienting of visual attention have already been tested, but there are many other behavioral instances of cognitive control. For example, humans can control whether to respond quickly but rather inaccurately, or slowly but accurately (Rinkenauer, Osman, Ulrich, Müller-Gethmann, & Mattes, 2004). Can such shifts along the speed-accuracy function be prompted subliminally by explicit cues? Likewise, participants in dual task situations can give priority to one or the other task (Pashler, 1984). Can such task prioritization be cued subliminally?

2. The second question relates to the reasons why unconscious events sometimes fail to prompt control, specifically when these prompts suggest the need of control implicitly. It is possible that there are conditions that prevented the discovery of implicit control, although it is possible in principle. For example, there might be adaptation to unconsciously induced response conflict, but sometimes the subliminally induced conflict was too small. There might also be adaptation to previous errors (post-error slowing), but perhaps in those cases where adaptation to unconscious errors was not found (e.g., van Gaal et al., 2009) errors were not sufficiently relevant for the participants. Finally, there might be an adaptation to unconsciously presented imbalances of conflict frequencies. However, these imbalances must be larger than those that are experienced consciously, or participants must be exposed to them much longer than to consciously perceived frequency imbalances.

Finally, we must be aware of the methodical problems that research on awareness faces from the beginning. First, effects of unconscious stimuli are often small. Therefore, studies aiming to show such effects must have sufficient power to do so. Second, research on the consciousness-control link is perhaps particularly susceptible to publication bias. Positive evidence for control without awareness is exciting and may make it easier to be published in prestigious journals (or to be published at all), while negative evidence resides in less prestigious journals (or may not be published). To justify such an intuition, of course, meta-analytical tools are needed (cf. Van den Bussche, Van den Noortgaate, & Reynvoet, 2009). Third, we must remain cautious regarding “indirect” consciousness-mediated explanations of apparently unconscious control effects. Masked events as such may remain unconscious, but their side-effects in behavior become apparent by self-observation. For example, the proportion of incongruent masked primes may remain undetected, but the resulting increase of error rates may be noticed and may prompt a corresponding adjustment indirectly (cf. Jaśkowski et al., 2003).

These problems are certainly not intractable, and they should definitely not prevent us from studying the important issue of the role of consciousness for cognitive control.
